# A microbiota-based perspective on urinary stone disease: insights from 16S rRNA sequencing and machine learning models

**DOI:** 10.3389/fcimb.2025.1623429

**Published:** 2025-10-23

**Authors:** Yufeng Liu, Aoyu Yang, Ziyi Zhang, Chen Shen, Wei Wang, Xiancheng Li

**Affiliations:** Department of Urology, The Second Affiliated Hospital of Dalian Medical University, Dalian, China

**Keywords:** calcium oxalate stones, uric acid stones, infectious stones, gut microbiota, urinary microbiota, machine learning models

## Abstract

**Background:**

Urinary stones are a multifactorial disease. In recent years, the role of microorganisms in its pathogenesis has attracted considerable attention. Although studies have suggested that certain microbes present in the gut and urine are associated with the formation of urinary stones, the current criteria for stone classification are not rigorous enough. Therefore, this study aimed to analyze the gut and urinary microbiota composition via 16S rRNA sequencing in patients with pure CaOx, pure UA, and pure Inf stones. By integrating these microbiota data with clinical data, we constructed machine learning models and evaluated their diagnostic value in distinguishing stone types.

**Methods:**

A total of 81 patients with urinary stones (including 30 with pure CaOx stones, 31 with pure UA stones, and 20 with pure Inf stones) and 26 healthy volunteers were enrolled. Stool and urine samples were collected from each participant and subjected to 16S rRNA sequencing to obtain microbiota data and characterize the gut and urinary microbiota profiles of patients with different stone types. We further integrated microbiota and clinical data, such as age, gender and BMI, using LASSO feature selection and six machine learning algorithms (e.g. SVM, Random Forest and XGBoost) to create prediction models for stone type. Model performance was evaluated through cross-validation.

**Results:**

Results showed enrichment of *Paramuribaculum*, *Muribaculum*, *Mesorhizobium*, and *Acinetobacter* in the gut of CaOx stone patients, with concurrent urinary enrichment of *Enterococcus*. Patients with UA stones demonstrated an increase in the abundance of *Massilioclostridium* in the gut and an increase in the abundance of *Fenollaria*, *Anaerococcus*, *Enterococcus* and *Escherichia* in the urine. Patients with Inf stones showed no differentially abundant gut taxa compared to healthy volunteers, but did exhibit urinary enrichment of *Escherichia*. The predictive model, which was based on urinary microbiota and clinical data, demonstrated excellent performance. The AUC was 0.922, 0.866 and 0.913 for the SVM, Random Forest and XGBoost models, respectively.

**Conclusion:**

This study reveals that different types of stone are characterized by distinct compositions of microbiota. Machine learning models based on microbiota and clinical data can predict urinary stone types noninvasively. This provides novel insights into the microecological mechanisms of urinary stones and opens up new avenues for clinical diagnosis.

## Introduction

Urinary stones are a multifactorial disease whose prevalence is influenced by a number of factors, including age, gender, diet, fluid intake, climatic conditions and ethnic differences (Scales et al., 2016; Liu et al., 2018; Wu et al., 2023). In recent years, the role of microorganisms in the formation of urinary stones has attracted increasing attention in the scientific community. It has been established that the presence of urease-producing bacteria in the urine can facilitate the breakdown of urea, thereby contributing to the alkalization of the urine (Tamborino et al., 2024). This, in turn, creates favorable conditions for the formation of infectious (Inf) stones (Tamborino et al., 2024). Furthermore, non-urease-producing bacteria have been demonstrated to possess the capacity to facilitate the growth and aggregation of calcium oxalate (CaOx) crystals (Chutipongtanate et al., 2013; Li et al., 2025).

CaOx stones are the most prevalent type of stone and their formation is closely related to urinary oxalate excretion. *Oxalobacter formigensis* is a pivotal species in the degradation of oxalate (Dawson et al., 1980; Allison et al., 1985; Daniel et al., 2021). However, subsequent studies have revealed that its efficacy in reducing urinary oxalate excretion is not consistent. The colonization rate is found to be influenced by a number of factors (Stewart et al., 2004; Hoppe et al., 2011; Daniel et al., 2021). Furthermore, it has been demonstrated that *Lactobacillus* and *Bifidobacterium* can also degrade oxalate (Abratt and Reid, 2010). This finding indicates that the association between bacteria and stones is extensive and encompasses a wide range of bacterial species, rather than being confined to a specific type of bacterium.

The advent of high-throughput sequencing technologies has provided deeper insight into the composition of the gut and urinary microbiota. Studies have shown that patients with urinary stone exhibit “ecological dysbiosis” in both the gut and urinary microbiota (Zampini et al., 2019; Zhao et al., 2021).

Nevertheless, current studies are limited by the lack of a precise classification based on stone composition. Therefore, this study used 16S rRNA sequencing to analyze the composition of the gut and urinary microbiota of patients with three different types of urinary stone. The aim was to shed light on the potential roles of gut and urinary microorganisms in the development of different types of stone. Furthermore, we developed machine learning models by combining microbiota and clinical data to assess their potential for differentiating stone types.

## Methods

### Study population and sample collection

At the Second Affiliated Hospital of Dalian Medical University in China, we recruited 81 patients with urinary stones, including 30 patients with CaOx stones (20 males, 10 females, mean age of 57.73 ± 11.13 years), 31 patients with uric acid (UA) stones (14 males, 17 females, mean age of 57.45 ± 12.03 years), and 20 patients with Inf stones (4 males, 16 females, mean age of 53.15 ± 12.46 years). Each patient had only one stone component, or the total composition of a single stone type was 100%. The composition of the stone components was obtained by Fourier transform infrared spectroscopy analysis. In addition, we recruited 26 healthy volunteers who were confirmed to be free of urinary stones via urinary ultrasound to serve as the control group (14 males and 12 females, mean age of 28.62 ± 2.90 years). The study was approved by the Ethics Committee of the Second Affiliated Hospital of Dalian Medical University in China, and all participants signed an informed consent form at the time of inclusion. Participants were excluded if they: (1) had used antibiotics within the past 4 weeks; (2) had inflammatory bowel disease, cystic fibrosis, celiac disease, chronic diarrhea, or a history of cancer; (3) had undergone bariatric surgery; (4) had a history of diabetes insipidus over the past year; (5) had urinary diversion or required indwelling urinary catheters or intermittent catheterization. And healthy volunteers with no history of urinary stones were included. Samples were collected into special containers and stored in a refrigerator at -80°C until DNA extraction.

### DNA extraction and PCR amplification

Microbial DNA was extracted from stool and urine samples according to the E.Z.N.A.^®^ Soil DNA Kit (Omega Bio-tek, Norcross, GA, USA) according to manufacturer’s protocols. The V1-V9 region of the bacteria 16S ribosomal RNA gene was amplified by PCR (95°C for 2 min, followed by 27 cycles at 95°C for 30 s, 55°C for 30 s, and 72°C for 60 s and a final extension at 72°C for 5 min) using primers 27F 5’-AGRGTTYGATYMTGGCTCAG-3’ and 1492R 5’-RGYTACCTTGTTACGACTT-3’, where barcode is an eight-base sequence unique to each sample (Pacific Biosciences, PN: 102-135-500). PCR reactions were performed in triplicate 20 μL mixture containing 4 μL of 5×FastPfu Buffer, 2 μL of 2.5 mM dNTPs, 0.8 μL of each primer (5 μM), 0.4 μL of FastPfu Polymerase, and 10 ng of template DNA. A negative control (using sterile water instead of template DNA) was included in each PCR run to check for potential contamination. Amplicons were extracted from 2% agarose gels and purified using the AxyPrep DNA Gel Extraction Kit (Axygen Biosciences, Union City, CA, U.S.) according to the manufacturer’s instructions.

### Library construction and sequencing

SMRTbell libraries were prepared from the amplified DNA by blunt-ligation according to the manufacturer’s instructions with SMRTbell prep kit 3.0 (Pacific Biosciences, PN: 102-182-700). Purified SMRTbell libraries from the pooled and barcoded samples were sequenced on a single PacBio Sequel IIe cell.

### Processing of sequencing data

PacBio raw reads were processed using the SMRT Link Analysis software version 11.0 to obtain demultiplexed circular consensus sequence (CCS) reads with the following settings: minimum number of passes=3, minimum predicted accuracy=0.99. Raw reads were processed through SMRT Portal to filter sequences for length (<1000 or >1800 bp) and quality. Sequences were further filtered by removing barcode and primer sequences with lima pipeline (Pacific Biosciences demultiplexing barcoded software, https://lima.how/).

After an average of 30,000 high-quality reads per sample were retained, chimeric sequences were identified and removed using UCHIME, followed by OTU clustering with a 98.65% similarity cutoff using UPARSE (version 10, http://drive5.com/uparse/). The phylogenetic affiliation of each 16S rRNA gene sequence was analyzed by uclust algorithm (https://github.com/topics/uclust) against the Silva (SSU138.2) 16S rRNA database (http://www.arb-silva.de) using confidence threshold of 80%.

### Statistical analysis and bioinformatics analysis

Statistical analyses were performed using SPSS 25.0 software. The measurement data were expressed as mean ± standard deviation and were compared using one-way analysis of variance or corrected one-way analysis of variance depending on normality and homogeneity of variance. The count data were expressed as the number of cases or percentage and were compared using the chi-square test of Fisher’s exact test when expected frequencies were low. The difference was considered to be statistically significant at *P* < 0.05.

The rarefaction analysis based on Mothur v.1.21.1 was conducted to reveal the diversity indices, including the Chao1, ACE, Simpson, and Shannon diversity indices (Li et al., 2013). An evaluation of beta diversity among four distinct groups was conducted through the implementation of principal coordinate analysis (PCoA), Adonis test, and ANOSIM test (Wei et al., 2019; Xiong et al., 2020). Wilcoxon rank-sum tests was employed to identify differentially abundant species, with false discovery rate (FDR) adjustment applied to Wilcoxon comparisons (Mann and Whitney, 1947).

### Machine learning models

The model was developed by combining species-level relative abundance data of gut and urinary microbiota with clinical data such as age, sex, and body mass index (BMI). Prior to model training, feature selection was performed using the LASSO (Least Absolute Shrinkage and Selection Operator) algorithm to reduce dimensionality and eliminate redundant features. The penalty coefficient alpha of the LASSO algorithm was optimized via intra-training-fold cross-validation, and the direction of key features selected by this algorithm was consistent with the results of differential abundance analysis. After feature filtering with LASSO, the finally retained features were incorporated into six classic supervised learning classifiers-AdaBoost, Support Vector Machine (SVM), Gradient Boosting, Extra Trees, Random Forest, and XGBoost-for the construction of multi-class classification models. These models were used to discriminate among the four groups: CaOx, UA, Inf, and control. For data splitting and validation, given the moderate sample size (total n=107) and class imbalance (30 cases in CaOx, 31 in UA, 20 in Inf, and 26 in control), the dataset was first randomly partitioned into an 80% training set and a 20% independent test set. Stratified 10-fold cross-validation was implemented on the training set to optimize the LASSO penalty coefficient and compare classifier performance. Stratified sampling was adopted in both data partitioning and cross-validation to preserve the proportional distribution of the four groups and address class imbalance during the sampling phase. The diagnostic performance of the models was evaluated by plotting Receiver Operating Characteristic (ROC) curves and calculating the Area Under the ROC Curve (AUC). Two complementary AUC metrics were reported to account for class imbalance: micro-averaged AUC, which is weighted by class frequency and reflects the overall model performance; and macro-averaged AUC, which is the unweighted mean of class-wise AUC values and emphasizes consistent performance across groups. The 95% confidence intervals were calculated for all AUC values.

## Results

### Subject characteristics

A total of 81 patients with urinary stones and 26 healthy volunteers were included in this study. Statistically significant differences were observed among the four groups with regard to age, gender and BMI (*P* < 0.001; *P* = 0.012; *P* < 0.001; **Table 1**).

**Table 1:** Characteristics of subjects.

Characteristic	CaOx	UA	Inf	Control	*P*
Age (years)	57.73 (11. 13)	57.45 (12.03)	53. 15 (12.46)	28.62 (2.90)	<0.001
Gender					0.012
Male	20	14	4	14	
Female	10	17	16	12	
BMI	24.56 (2.66)	27.08 (4. 13)	25.89 (4. 10)	23.02 (3. 19)	<0.001

### Diversity of the microbiota

**Figure 1:**
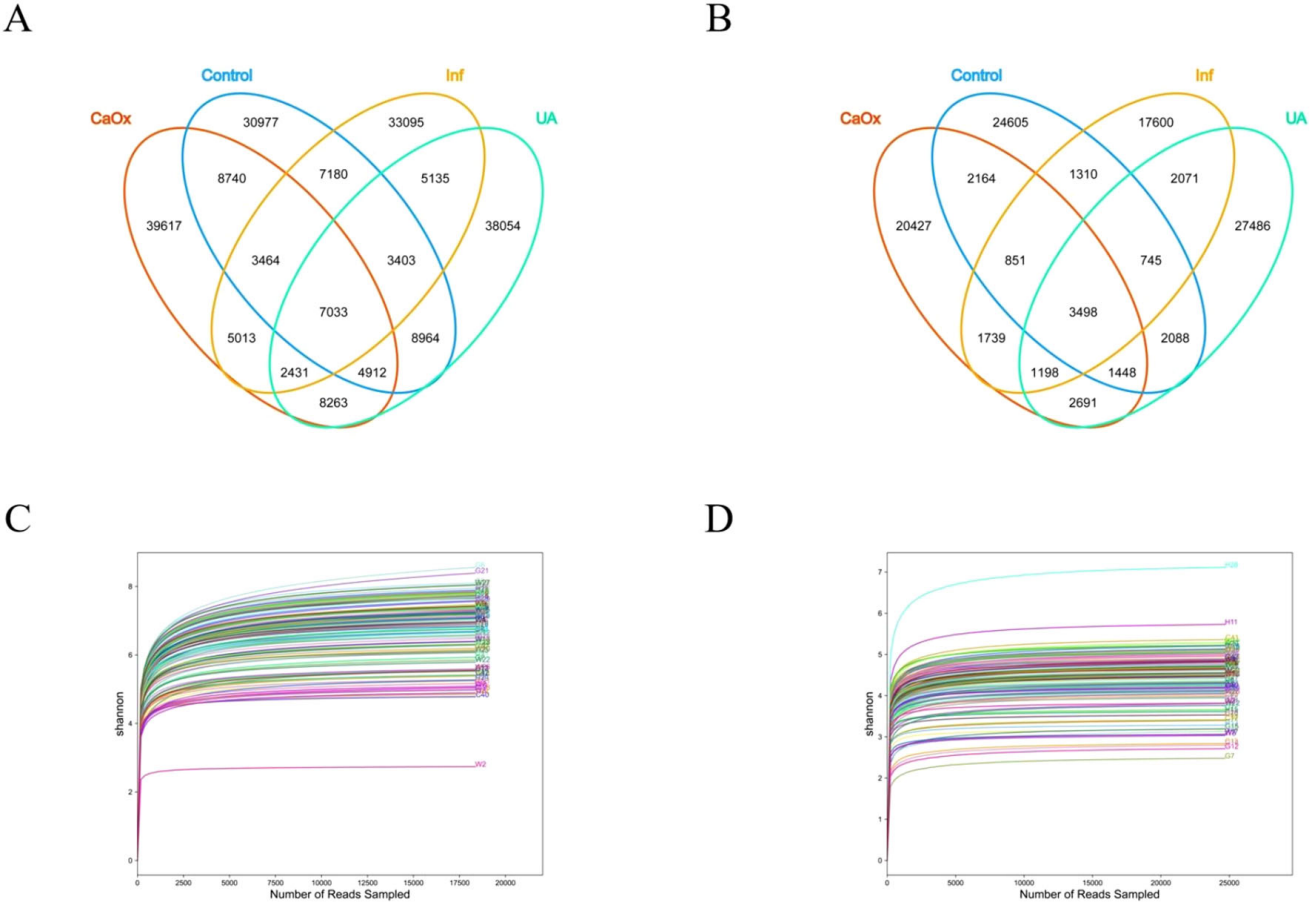
Analysis of gut and urinary microbiota in each group using 16S rRNA. **(A)** Venn diagram showing the shared and unique OTUs in the gut among the four groups. **(B)** Venn diagram showing the shared and unique OTUs in the urine among the four groups. **(C)** Shannon-Wiener curve: used to reflect the diversity of the gut microbiota. **(D)** Shannon-Wiener curve: used to reflect the diversity of the urinary microbiota.

To characterize the microbiota between the stone and control groups, we performed 16S rRNA sequencing on all subject samples and obtained 206,281 OTUs in the gut and 109,921 OTUs in the urine (**Figures 1A, B**). A total of 778 genera were identified in the gut and 1,242 genera were identified in the urine (**Supplementary Table 1**). As the Shannon-Wiener curves gradually flattened, this indicated that the applied sequencing depth had sufficiently covered the biodiversity (**Figures 1C, D**).

**Figure 2:**
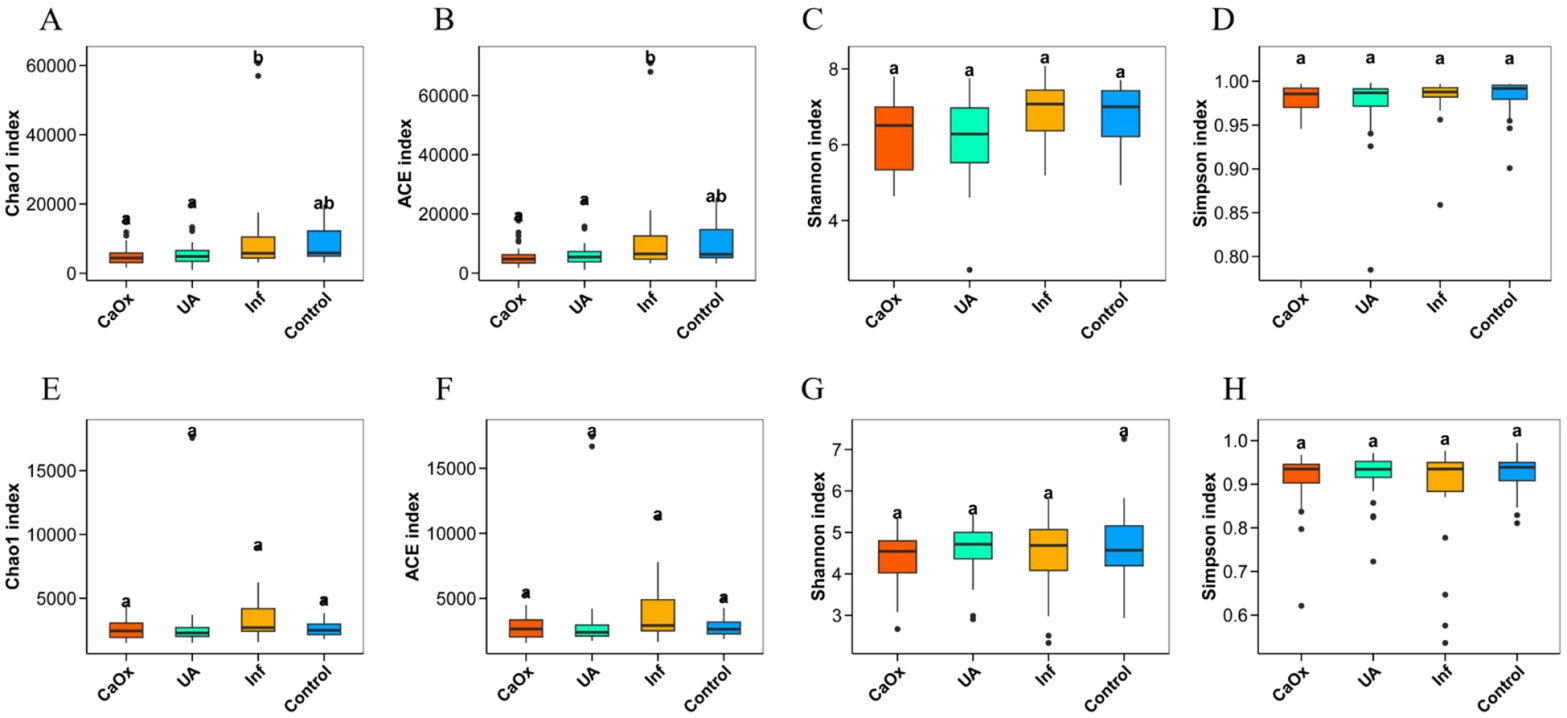
Comparison of alpha diversity of gut microbiota and urinary microbiota among groups ACE and Chao1 are indices that estimate the number of OTUs contained in the sample; Shannon and Simpson are indices that estimate the microbial diversity in the sample. **(A–D)** Alpha diversity indices for gut microbiota. **(E–H)** Alpha diversity indices for urinary microbiota.

Microbial alpha diversity was described by four indices: Chao1, ACE, Shannon and Simpson. Significant differences were observed in Chao1 and ACE indices, which are used to assess gut microbial alpha diversity, in comparisons of CaOx vs. Inf groups and UA vs. Inf groups. However, no significant differences were detected in alpha diversity indices of either gut or urinary microbiota when comparing each of the three stone groups with the control group (**Figures 2A–H**).

**Figure 3:**
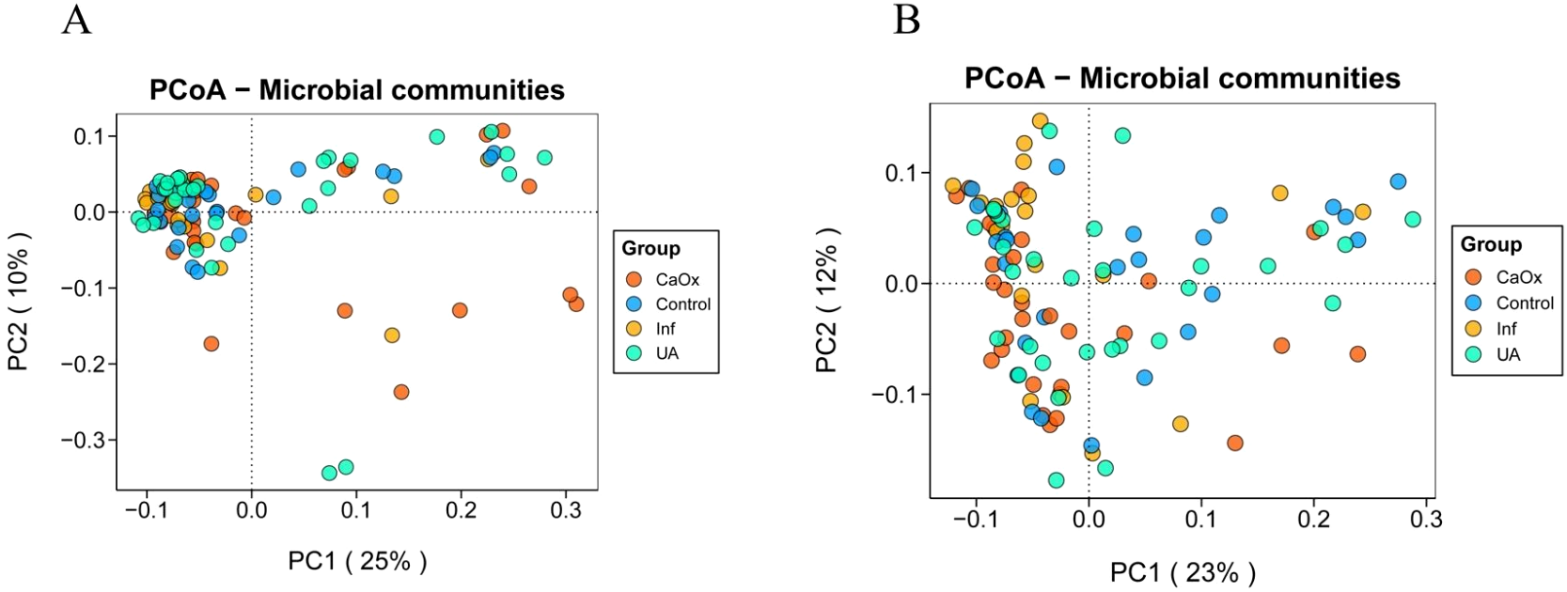
Beta diversity of gut and urinary microbiota among the four groups. **(A)** Bray-Curtis distance PCoA plot for the gut microbiota. **(B)** Bray-Curtis distance PCoA plot for the urinary microbiota.

PCoA analysis revealed significant differences in beta diversity among the four groups, although there was no clear separation (**Figures 3A, B**). Statistical analysis revealed that significant differences were detected among the four groups based on Bray-Curtis distances as assessed by Adonis (*P* = 0.001; *P* = 0.018). It was found that the Bray-Curtis distances that passed the ANOSIM test were also statistically different (*P* = 0.006; *P* = 0.021).

### Differentially abundant taxa

**Figure 4:**
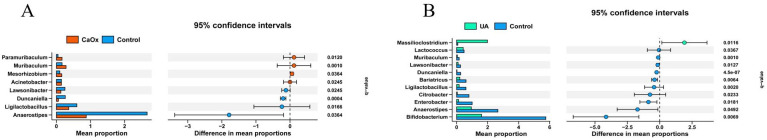
Wilcoxon rank-sum tests after FDR correction. Bars show the relative abundance of CaOx and UA that differ at the genus level. **(A)** Gut microbiota of patients with CaOx stones. **(B)** Gut microbiota of patients with UA stones.

**Figure 5:**
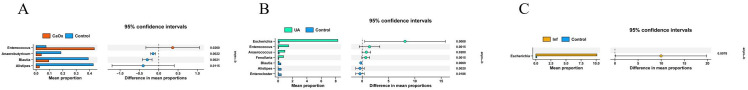
Wilcoxon rank-sum tests after FDR correction. Bars show the relative abundance of CaOx, UA and Inf that differ at the genus level. **(A)** Urinary microbiota of patients with CaOx stones. **(B)** Urinary microbiota of patients with UA stones. **(C)** Urinary microbiota of patients with Inf stones.

To further clarify regarding the microbiota composition differences among the three stone groups and the control group, a comparison of taxa at the genus level was performed using the Wilcoxon rank-sum test. The test results were adjusted for the FDR. When compared to the control group, patients with CaOx stones exhibited significant enrichment of four taxa in the gut; those with UA stones had one significantly enriched taxon; and no differentially abundant taxa were detected in the gut of Inf stone patients (**Figures 4A, B**; **Supplementary Table 2**). One taxon was significantly enriched in the urine of CaOx stone patients, four in UA stone patients, and one in Inf stone patients, respectively (**Figures 5A-C**; **Supplementary Table 3**).

### Machine learning models

**Figure 6:**
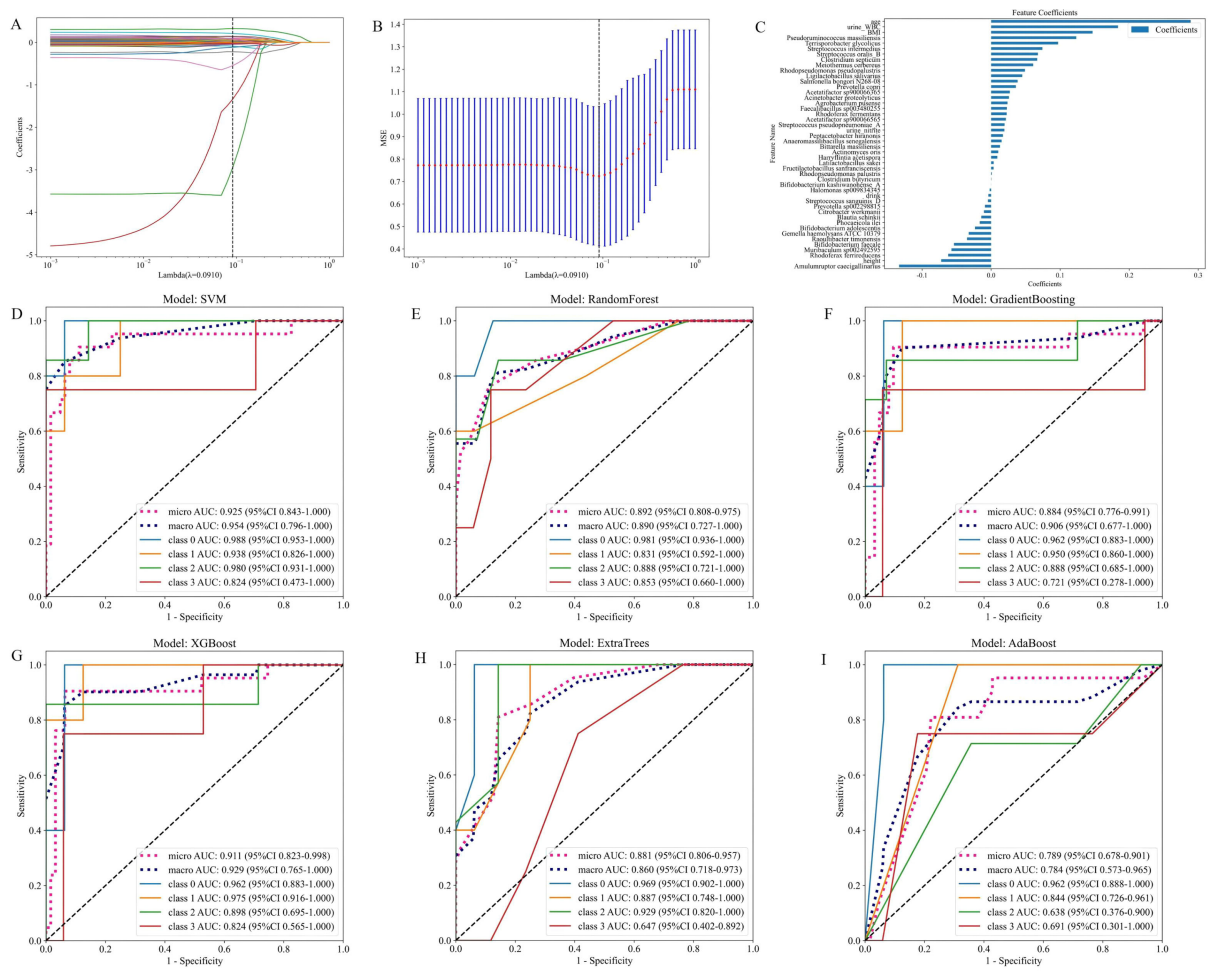
Models constructed by combining patient gut microbiota 16S strain sequencing results and subject characteristics. **(A)** Feature fusion selection based on the LASSO algorithm. **(B)** MSE and tenfold cross-validation coefficients and **(C)** histogram of feature scores based on selected characteristics. **(D-I)** ROC curves for the test set of six machine learning models.

As illustrated in **Figures 6A–C**, the model integrates patient gut 16S rRNA sequencing data with clinical data. It presents the fused feature selection based on the LASSO algorithm, the mean squared error (MSE) and 10-fold cross-validation coefficients, and a histogram of the feature scores for the selected characteristics. **Figures 6D–I** presents the ROC curves for six machine learning models (SVM, Random Forest, Gradient Boosting, XGBoost, Extra Trees and AdaBoost) evaluated on the cross-validation folds. Overall, the models achieved discriminative performance, with SVM, XGBoost, and Random Forest demonstrating relatively higher predictive accuracy (macro-AUC: 0.954, 0.890 and 0.929, respectively). The full set of micro-AUC, macro-AUC values, and their 95% confidence intervals for all models are provided in **Figure 6**.

**Figure 7:**
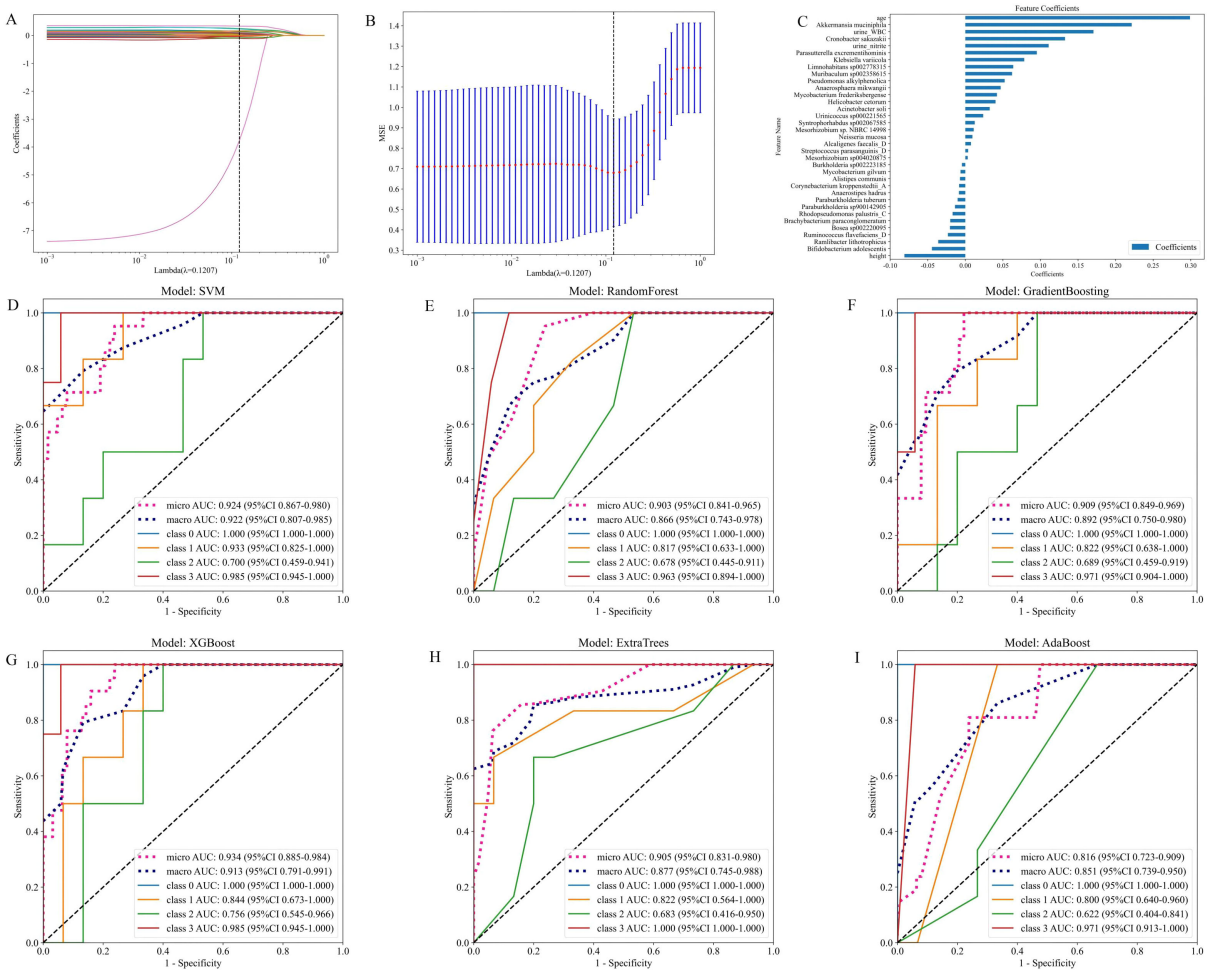
Models constructed by combining patient urinary microbiota 16S strain sequencing results and subject characteristics. **(A)** Feature fusion selection based on LASSO algorithm. **(B)** MSE and tenfold cross-validation coefficients and **(C)** Histogram of feature scores based on selected characteristics. **(D-I)** ROC curves for the test set of six machine learning models.

As illustrated in **Figures 7A–C**, the model integrates patient urine 16S rRNA sequencing data with clinical data. It presents the fused feature selection based on the LASSO algorithm, the MSE and 10-fold cross-validation coefficients, and a histogram of the feature scores for the selected characteristics. **Figures 7D–I** presents the ROC curves for the six machine learning models, evaluated on the cross-validation folds. Overall, the models achieved favorable discrimination performance. The SVM, Random Forest and XGBoost models demonstrated the higher predictive accuracy (macro-AUC values of 0.922, 0.866 and 0.913, respectively). The full set of micro-AUC, macro-AUC values, and their 95% confidence intervals for all models are provided in **Figure 7**.

## Discussion

This study analyzed the gut and urinary microbiota in patients with CaOx stones, UA stones, and Inf stones, revealing distinct patterns of microbial community dysbiosis specific to each stone type. It is noteworthy that the prediction model based on urinary microbiota and clinical data demonstrated superior discriminative ability compared to the gut microbiome-based model, highlighting its potential value for distinguishing among different stone types.

The results of this study indicate that *Paramuribaculum*, *Muribaculum*, *Mesorhizobium*, and *Acinetobacter* were enriched in the gut of CaOx stone patients. Both *Paramuribaculum* and *Muribaculum* are members of *Muribaculaceae* (Smith et al., 2021; Zhu et al., 2024). Previous studies have reported the capacity of bacterial strains belonging to this family to degrade oxalate. The enrichment of these bacteria in these patients may be substrate-driven. These individuals often have increased intestinal oxalate absorption or higher dietary oxalate intake, leading to elevated intestinal luminal oxalate concentrations that create favorable conditions for bacterial growth and proliferation (Miller et al., 2016). Although the role of *Mesorhizobium* is well-established in the context of plant symbiosis, its function within the human gut remains unclear (Wang et al., 2022). Its enrichment in the patient’s gut may suggest the emergence of a specific ecological niche under the condition of gut microbiota dysbiosis. It is recommended that subsequent studies examine whether its enrichment in the gut has a substantial impact on the development of CaOx stones. *Acinetobacter*, a prevalent opportunistic pathogen, can trigger an inflammatory response and compromise intestinal barrier integrity. This, in turn, may increase paracellular oxalate absorption, thereby driving the initiation and progression of CaOx stones (Doughari et al., 2011). *Enterococcus*, which is enriched in the urine of CaOx stone patients, relies on purine and carbohydrate metabolism. This suggests that it may influence the crystallization microenvironment of the stones by altering the chemical composition of urine (Tan et al., 2024). Furthermore, *Enterococcus* has the ability to form biofilms. These biofilms have the capacity to entrap mineral ions and crystals in the urine, thereby accelerating the processes of crystal formation and aggregation. Concurrently, *Enterococcus* has been observed to induce damage to the urothelial mucosa and upregulate the expression of factors such as osteopontin. This enhances the retention capacity of crystals in the urine, collectively promoting the initiation and progression of CaOx stones (Fisher and Phillips, 2009; Arias and Murray, 2012; Djojodimedjo et al., 2013; Nicu-Canareica et al., 2025).

This study found that *Massilioclostridium* was enriched in the gut of patients with UA stones. Only a limited number of publications have reported a potential association between this genus and human diseases, and its biological function requires further elucidation (Wang et al., 2024). *Fenollaria*, *Anaerococcus*, *Enterococcus*, and *Escherichia* were enriched in the urine of patients with UA stones. *Fenollaria* and *Anaerococcus* have been reported to decrease urinary pH, thereby creating favorable conditions for the formation of UA stones (Abou-Elela, 2017; Hurst et al., 2024). *Fenollaria* sp*orofastidiosus* has been reported to encode a predicted citrate lyase complex, which is involved in the citrate degradation pathway and metabolizes citrate to oxaloacetate and acetyl-CoA (Schwaderer and Wolfe, 2017; Segall et al., 2024; Skolarikos et al., 2024). Low systemic citrate levels have been demonstrated to compromise its regulatory capacity on urinary pH, thereby potentially increasing the risk of UA stone formation. *Enterococcus* and *Escherichia* can release ATP via lysosomal exocytosis. This ATP is then enzymatically converted to urate,thereby promoting the formation of UA stones (Silberfeld et al., 2020).

While no differentially abundant taxa were identified in the gut of Inf stone patients compared to healthy volunteers, *Escherichia* was enriched in the patients’ urine. Previous studies have demonstrated that it possesses urease activity, which catalyzes urea hydrolysis to generate ammonia and carbon dioxide. This reaction elevates urinary pH, thereby promoting the formation of Inf stones such as magnesium ammonium phosphate (struvite) and carbonate apatite (Halinski et al., 2022; Skolarikos et al., 2024). These findings underscore the predominant role of urinary microbial activities and the host urinary environment in the pathogenesis of Inf stones. While the gut microbiota plays crucial roles in overall metabolism and immune regulation, its direct contribution to Inf stone formation appears to be more limited, which could explain the absence of gut microbial differences observed in our analysis.

Preoperative identification of the primary stone composition is of guiding significance for clinical decision-making. However, conventional imaging modalities, such as CT or urinary ultrasound, lack the capability to accurately determine the specific stone type prior to surgery. A meta-analysis revealed significant differences in microbiota diversity when stratified by the presence of urolithiasis, stone composition, age, and study location. Furthermore, the OTU-based taxonomic approach exhibited superior performance in distinguishing sample types and gender. These findings emphasize the potential of microbiota in predicting stone types (Kachroo et al., 2021). Microbiota-based machine learning has shown promising performance for diagnosing both juvenile idiopathic arthritis and neonatal jaundice (Chen et al., 2024; Tu et al., 2024). The integration of gut and urinary microbiota with clinical data (e.g. age, gender, metabolic indicators) was therefore undertaken in order to develop a clinical prediction model. The objective of this model is to predict stone types and compare the performance of the two groups, thereby enhancing its predictive power. The integration of multimodal data holds superior diagnostic value.

In this study, comparing the CaOx stone, UA stone, Inf stone, and control groups. The predictive model we constructed demonstrated superior performance among the six machine learning models. Studies have shown that, compared to the gut microbiota, the urinary microbiota can more accurately distinguish between stone formers and non-stone-formers (Zampini et al., 2019). In our study, the predictive model integrating urinary microbiota with clinical data outperformed the model based on gut microbiota, which is consistent with previous findings. Additionally, our model demonstrated superior efficacy compared to the machine learning model that combined gut microbiota and clinical data for predicting CaOx stone formation (Xiang et al., 2022).

Our research presents the first systematic classification of urinary stones, moving beyond the traditional binary comparison (stone-formers vs. non-stone-formers) or rough subtyping by composition. Such a refined classification strategy facilitates the acquisition of more pathologically specific microbial profiles. Secondly, we analyzed the microbiota from both gut and urine and compared their effectiveness in discriminating stone types. The model that combined urinary microbiota and clinical data exhibited superior predictive performance. This provides key evidence for the development of non-invasive diagnostic tools. Furthermore, we integrated microbiota and clinical data, employing multiple machine learning algorithms to construct predictive models. This demonstrates the considerable potential of multimodal data fusion for accurately discriminating among stone types. These systematic explorations offer fresh insights into the microbial contributions to stone formation, thereby establishing the methodological groundwork for creating future microbiota-targeted therapies and auxiliary diagnostic instruments.

The main limitation of this study was the restricted sample size resulting from its single-center design. Particularly in the Inf stone group (n=20), the small cohort may affect statistical power and limit the generalizability of the findings. Furthermore, significant differences in demographic characteristics (e.g., age, gender, and BMI) existed between the control and stone groups. These differences may have a confounding effect on the structure of the microbiota, making it difficult to attribute the observed microbial variations solely to the disease itself. Additionally, the absence of dietary assessments via food questionnaires prevents the exclusion of dietary influences. Compounding this issue, antibiotic usage was self-reported, which may introduce bias into the results. Methodologically, this study assessed the relative abundance of microbiota via 16S rRNA sequencing rather than absolute abundance. In future research, quantitative PCR (qPCR) could be employed to provide a more precise quantitative analysis. The modest sample size for the machine learning models suggests that the reported AUC values of 0.89–0.95 may be subject to bias. These performance metrics were derived solely from internal validation, including data partitioning and K-fold cross-validation within the same dataset. This approach poses a risk of overfitting and limits the generalizability of the model, as it does not involve independent external cohort validation. Finally, due to the observational design of this study, we cannot infer a causal relationship between the microbiota and urinary stone formation.

Building on the initial insights into the microbiota profiles of urinary stone patients revealed by this study, large-scale multicenter cohort studies should be undertaken in the future. In these studies, rigorous matching or statistical adjustment for key confounders such as age, gender, and BMI during participant recruitment is necessary to validate the observed microbiota-stone type associations and enhance the generalizability of the findings. And future work integrating metagenomics, metabolomics, and animal models is essential to functionally validate the putative mechanisms uncovered in this study and to establish causal links between the microbiota and urinary stone formation.

## Conclusion

In summary, this study confirmed significant associations between stone types and distinct patterns of microbiota dysbiosis by analyzing the gut and urine microbiota of patients with different types of urinary stone. Specifically, microorganisms enriched in patients with CaOx stones are implicated in the disease process via multiple mechanisms, such as affecting gut barrier integrity and modifying urinary environment. For UA stones, relevant dysbiosis may function through altering urine pH and promoting urate generation. Meanwhile, Inf stones are definitively driven by enriched urease-producing microorganisms (e.g., *Escherichia*) that induce an alkaline milieu. These findings suggest that urinary stone formation and progression are closely linked to structural and functional disruptions to the human microbial ecosystem. Therefore, incorporating the microbiota into the pathophysiological framework of urinary stones is crucial for deepening our understanding of its pathogenesis and developing novel microecological intervention strategies.

## Data Availability

Original datasets are available in a publicly accessible repository:The original contributions presented in the study are publicly available. This data can be found here: https://www.ncbi.nlm.nih.gov/, PRJNA1332921; PRJNA1332924.
