# Supplementary material for: A microbiota-based perspective on urinary stone disease: insights from 16S rRNA sequencing and machine learning models
Source: Front Cell Infect Microbiol. 2025 Oct 23;15:1623429. doi: 10.3389/fcimb.2025.1623429 (PMC12589065; doi:10.3389/fcimb.2025.1623429)
Supplement: Supplementary file 1 [file Table1.docx]

SUPPLEMENTARY TABLE 1 The number of features obtained at each taxonomic level after OTU annotation.

| **Sample type** | **Phyla** | **Class** | **Order** | **Family** | **Genera** | **Species** |
| --- | --- | --- | --- | --- | --- | --- |
| Stool | 29 | 56 | 113 | 238 | 778 | 2053 |
| Urine | 42 | 72 | 151 | 342 | 1242 | 3643 |
