# Supplementary material for: A microbiota-based perspective on urinary stone disease: insights from 16S rRNA sequencing and machine learning models
Source: Front Cell Infect Microbiol. 2025 Oct 23;15:1623429. doi: 10.3389/fcimb.2025.1623429 (PMC12589065; doi:10.3389/fcimb.2025.1623429)
Supplement: Supplementary file 2 [file Table2.docx]

| **Group** | **Enriched taxa** | **Reduced taxa** |
| --- | --- | --- |
| CaOx | *Paramuribaculum;*  *Muribaculum;*  *Mesorhizobium;*  *Acinetobacter* | *Lawsonibacter;*  *Duncaniella;*  *Ligilactobacillus;*  *Anaerostipes* |
| UA | *Massilioclostridium* | *Lactococcus;*  *Muribaculum;*  *Lawsonibacter;*  *Duncaniella;*  *Bariatricus;*  *Ligilactobacillus;*  *Citrobacter;*  *Enterobacter;*  *Anaerostipes;*  *Bifidobacterium* |
| Inf | - | - |

SUPPLEMENTARY TABLE 2 Identification of gut taxa that were significantly enriched or reduced across stone patient groups, using Wilcoxon rank-sum tests with FDR adjustment for multiple comparisons.
