# Supplementary material for: A microbiota-based perspective on urinary stone disease: insights from 16S rRNA sequencing and machine learning models
Source: Front Cell Infect Microbiol. 2025 Oct 23;15:1623429. doi: 10.3389/fcimb.2025.1623429 (PMC12589065; doi:10.3389/fcimb.2025.1623429)
Supplement: Supplementary file 3 [file Table3.docx]

| **Group** | **Enriched taxa** | **Reduced taxa** |
| --- | --- | --- |
| CaOx | *Enterococcus* | *Anaerobutyricum;*  *Blautia;*  *Alistipes* |
| UA | *Escherichia;*  *Enterococcus;*  *Anaerococcus;*  *Fenollaria* | *Blautia;*  *Alistipes;*  *Enterocloster* |
| Inf | *Escherichia* | *-* |

SUPPLEMENTARY TABLE 3 Identification of urine taxa that were significantly enriched or reduced across stone patient groups, using Wilcoxon rank-sum tests with FDR adjustment for multiple comparisons.
